# Supplementary material for: SV-plaudit: A cloud-based framework for manually curating thousands of structural variants
Source: Gigascience. 2018 May 31;7(7):giy064. doi: 10.1093/gigascience/giy064 (PMC6030999; doi:10.1093/gigascience/giy064)
Supplement: GIGA-D-18-00103_Revision_1.pdf [file giy064_giga-d-18-00103_revision_1.pdf]

GigaScience

SV-plaudit: A cloud-based framework for manually curating thousands of structural variants

--Manuscript Draft--

**Manuscript Number:**	GIGA-D-18-00103R1
**Full Title:**	SV-plaudit: A cloud-based framework for manually curating thousands of structural variants
**Article Type:**	Research
**Funding Information:**	National Human Genome Research Institute (K99HG009532) — Dr Ryan Layer<br>National Human Genome Research Institute (R01HG006693) — Dr Aaron R Quinlan<br>National Human Genome Research Institute (R01GM124355) — Dr Aaron R Quinlan<br>National Cancer Institute (US) (U24CA209999) — Dr Aaron R Quinlan
**Abstract:**	SV-plaudit is a framework for rapidly curating structural variant (SVs) predictions. For each SV, we generate an image that visualizes the coverage and alignment signals from a set of samples. Images are uploaded to our cloud framework where users assess the quality of each image using a client-side web application. Reports can then be generated as a tab-delimited file or annotated VCF. As a proof of principle, nine researchers collaborated for one hour to evaluate 1,350 SVs each. We anticipate that SV-plaudit will become a standard step in variant calling pipelines and the crowd-sourced curation of other biological results.
**Corresponding Author:**	Ryan Layer<br><br>UNITED STATES
**Corresponding Author Secondary Information:**	
**Corresponding Author's Institution:**	
**Corresponding Author's Secondary Institution:**	
**First Author:**	Jonathan R Belyeu
**First Author Secondary Information:**	
**Order of Authors:**	Jonathan R Belyeu
	Thomas J Nicholas, PHD
	Brent S Pedersen, PHD
	Thomas A Sasani
	James M Havrilla
	Stephanie N Kravitz
	Megan E Conway
	Brian K Lohman, PHD
	Aaron R Quinlan, PHD
	Ryan Layer
**Order of Authors Secondary Information:**	

Powered by Editorial Manager® and ProduXion Manager® from Aries Systems Corporation

Response to Reviewers:	Below are our point-by-point responses to all of issues raised by Reviewers 1 and 2. Our responses are wrapping in “----” markers.<br><br>Reviewer #1: The authors produce a tool that facilitates visual inspection of putative structural variants (i.e. deletions, inversions, duplications, insertions) based on reads mapped to a reference genome. The key innovation is that the software is set up so that a single researcher can rapidly visualize and categorize the existence of large numbers of putative structural variants. This enables a form of "crowd" evaluation such that every putative variant is visually inspected by multiple people. The software dramatically lowers the effort required to have manual inspection of manual curation of hundreds or even thousands of putative structural variants. This can lead to a strong increase in the reliability of putative SVs for downstream analyses and the development of new SV detection algorithms.<br><br>All the code is on Github with MIT license, the design of the software is modular for flexibility. This is pleasant.<br><br>I have not run the software, but the code and documentation appear to be functional, and the software uses standard input and output formats.<br><br>A weakness with the manuscript is that the software has only been tested on what the authors themselves call "the incredibly high quality" NA12878 genome in a bottle data (300x and PCR free), while also including the individual's parents. As the authors point out (L7-9), typical WGS datasets have been 30X coverage and with PCR-amplification during library preparation. There would thus be more power to evaluate the relevance of this software if PCR-biased, lower-coverage data were used (or simulated).<br><br>----<br>This is a good point. We have had success using SV-Plaudit on some internal sequencing experiments that were at 5X, 33X and 58X coverage. To help demonstrate this broader utility, we added Supplemental Figure 2 that shows an SV from NA12878 at these different coverage levels.<br>----<br><br>Some additional minor comments that could help to improve the manuscript & visualization:<br><br>1. the meaning of "GOOD" vs "BAD" vs "DE NOVO" is not immediately clear (e.g. L24 p3). And further appears to be at odds with the screenshots shown in the youtube video (Supports vs does not support vs de novo). Further more "de novo" is somewhat misleading as it suggest that something completely novel has occurred in the focal sample. Some efforts to make these buttons/meaning completely unambiguous would be justifiable. E.g., just have single statement: "Read mapping in the top image indicate that the sample has a xxx yyyy (e.g. 248bp DELETION) compared to the reference genome", then "TRUE", "FALSE" or "There appears to be a structural variant, but it differs from your suggestion". I also suspect that data could be cleaner if a fourth button existed to make it possible for users to say "I don't know".\<br><br>----<br>A strength of SV-Plaudit is that the “curation question” and “curation answers” are defined by the project manager, and one is free to easily customize the prompts to exactly fit your experiment. If there is a more efficient or less ambiguous way to prompt users, or if a third or fourth answer option is appropriate (e.g., a choice of “The region is too noisy” and “The region does not have adequate depth”), then one only needs to adjust the configuration file. We appreciate this feedback, as we do not think that we made this point clearly or strongly enough. While we discussed the details of how to customize the questions in the PlotCritic section of the methods, we also added more text addressing this issue in the discussion and background.<br>----<br><br>2. The manuscript takes putative SVs detected by the 1000 genomes project, evaluates them using SVTYPER users and then compares the results to those obtained using SVTYPER and CNVATOR. I suspect that SVTYPER and/or CNVATOR may have been used to create the initial putative SV dataset during the 1000G project.

Powered by Editorial Manager® and ProduXion Manager® from Aries Systems Corporation

In which case this would be some circularity. A commentary on this would be welcome. Similarly, for those wanting to apply SVTYPER to a new genomic dataset, a recommendation on how to find putative SVs would be welcome.

This is not an issue that we considered and I thank the reviewer for bringing it to our attention. According to the 1000 genomes SV paper, CNVNATOR was used, and SVTYPER was not. Interestingly, the rate of false calls (false positives for CNVNATOR and false negatives for SVTYPER) was about the same for both methods (44.2% for CNVATOR and 30.7% for SVTYPER). When we go back and look at which algorithms were used in CNVNATOR's false positives, they were all made by either a union of callers or by one of the other nine methods. We have added this commentary to the discussion of these results because it is another example of how every SV caller has its strengths and weaknesses, and why we believe visual validation is important.

As for adding text to the manuscript about using SVTYPER on a new dataset, we do not feel like we have the data to go beyond noting that in our experiment SVYTYPER marks some real deletions as homozygous reference. We hope that readers interpret the CNVNATOR and SVTYPER results as proof that it is difficult to rely on automated methods, and that visualization can help close the confidence gap for SVs.
----

3. Does PlotCritic have the option of hosting on a local machine, eg. using flask, instead of Amazon cloud? (for those with limited budgets, in places where AWS is difficult to access, and to cover for the situation where Amazon's API will change?)

This is a good point and we recognize this need. While Amazon hosting is all that is available right now, local hosting is actively being developed and will be available in a future release that we are planning for this year. Furthermore, Amazon provides an option to specify the API version desired for an application, which we use to maintain access and usability.
----

4. The screenshots and youtube video only appear to show DELETIONS. I would want to get a feeling for what duplications and insertions look like before using this software.

We agree that other SV types need to be shown and we have added Supplemental Figure 1, which includes a duplication and an inversion and updated the manuscript to refer to them.
----

5. Locations of read pair mappings may be clearer if there were no border on the pair of boxes and the line connecting the boxes were the same intensity as the boxes themselves (currently, the line goes from the middle of each square and is darker than the fill of the box)

We tried this and many other plot configurations, and we ultimately we decided that the current plots are most often the easiest to interpret. Thankfully the code is open source and advanced users can make small changes to the code to customize their plots. We have added comments in the code to make the appropriate lines easier to find and modify.
----

6. It took me a while to understand that the Y axis on each sample differed. Have you toyed with homogenising it? And/or perhaps showing it on a log scale?

Yes, we tried this and in our opinion it makes the plots less clear since the log transformation has the largest effect on the smallest insert size. We have also added the "--common_insert_size" option to samplot to use the same left y-scale across the

plots.
----

7. Legend of Fig 1 might want to explicitly mention that NA12891 and 2 are parents of 12878. Furthermore it may want to mention that the top one is the one being evaluated.

Thank you. We have added this text to the figure.

Reviewer #2:

The authors of the manuscript "SV-plaudit: A cloud-based framework for manually curating thousands of structural variants" propose a framework to easily manually assess if SVs are potentially false or true. This is enabled based on a cloud based pipeline, which allows to look at multiple thousand sites for a larger community. Overall I think that this is an important contribution for multiple projects such as GiaB or other where scientist need to assess the quality of their discovered SVs.

In the following some concerns and questions:

1. I am wondering if you could comment what had a deeper impact in the evaluation: a) the visualization or b) the ability to look at the trio

----
Unfortunately, we do not know how the experiment participants felt because we did not ask. We expect that visualizing a trio would have a large impact on identifying true and false variants. We developed the tool around multi-sample visualization so that the users could get a sense of an SV’s genomic context (e.g., is the area generally mess) from the control samples. A trio is helpful because users are able to observe both the genomic context and, in most cases, the inheritance of the SV.
----

2. I would encourage to include the mappability track of some kind (e.g. 36bp) to give the users more control and insight of the variability observed at the breakpoints. I know you stated that this needs to be part of a future research, but I think that is easy to obtain (UCSC) and integrate. Another maybe very useful feature would be the frequency of the reads that support the event.

This is a good point. Depending on the experiment, there are many types of annotations that users may want to see (e.g., repetitive elements, miRNAs, TAD boundaries). SAMPLOT has the option of displaying a gene annotation track. We have generalized this and exposed it to the SV-PLAUDIT configuration so that users can include any BED annotation (using the -A option) options.
----

3. I would encourage you to provide also figures for the other types of SVs not just Deletions. E.g. how do you visualize BND or other events?

We have added Supplementary Figure 1 that includes a duplication and an inversion, and BND visualization is under development.
----

4. I think your demonstration is really nice over the 1000 genomes data. What I would liked to see further is for some validated SVs if the figures are consistently clear. I know this is maybe out of the scope of this study, but maybe showing a few examples of the pass vs. non pass SVs from GiaB call set 0.5.0, which hopefully are close to the truth might give further insights on the reliability of the method. This is especially interesting since you mention false discovery and sensitivity issues over computational genotyping SVs.

Powered by Editorial Manager® and ProduXion Manager® from Aries Systems Corporation

	We have added Supplementary Figure 3 based on the GiaB 0.5.0 call set and the GiaB/NIST/NHGRI Illumina sequencing of the Ashkenazim trio. The figure contains four panels, two SVs (A and B) were labeled PASS and two (C and D) were not labeled as PASS (LongReadHomRef and NoConsensusGT). The visualizations match the validation status of the VCF file.<br>----<br><br>The latest GiaB SV results<br>5. I found Figure 1 A rather confusing since I only see the coverage. Is this due to the size of the region and thus the points on the bottom are the read pairs? In that case there should be some pairs that span the deletion, right? Could you maybe sort the reads better that support the SV, or more general show abnormal distances?<br><br>----<br>Figure 1A has a large cluster of both paired-end alignments (boxes and solid lines) and split-read alignments (circles and dashed lines) that appear to support the the SV in both NA12878 (top sample) and her dad. We have added some annotations to Figure 1A to make it easier to identify the different components.<br>----
**Additional Information:**	
**Question**	**Response**
Are you submitting this manuscript to a special series or article collection?	No
**Experimental design and statistics**<br><br><br>Full details of the experimental design and statistical methods used should be given in the Methods section, as detailed in our Minimum Standards Reporting Checklist. Information essential to interpreting the data presented should be made available in the figure legends.<br><br><br>Have you included all the information requested in your manuscript?	Yes
**Resources**<br><br><br>A description of all resources used, including antibodies, cell lines, animals and software tools, with enough information to allow them to be uniquely identified, should be included in the Methods section. Authors are strongly encouraged to cite Research Resource Identifiers (RRIDs) for antibodies, model organisms and tools, where possible.<br><br><br>Have you included the information requested as detailed in our Minimum Standards Reporting Checklist?	Yes
**Availability of data and materials**	Yes

All datasets and code on which the conclusions of the paper rely must be either included in your submission or deposited in publicly available repositories (where available and ethically appropriate), referencing such data using a unique identifier in the references and in the "Availability of Data and Materials" section of your manuscript.<br><br>Have you have met the above requirement as detailed in our Minimum Standards Reporting Checklist?	

Powered by Editorial Manager® and ProduXion Manager® from Aries Systems Corporation

Manuscript Click here to download Manuscript GigaScience_Revision.pdf

SV-plaudit: A cloud-based framework for manually curating thousands of structural variants

Jonathan R. Belyeu[1,2], Thomas J, Nicholas[1,2], Brent S. Pedersen[1,2], Thomas A. Sasani[1,2], James M. Havrilla[1,2], Stephanie N. Kravitz[1,2], Megan E. Conway[1], Brian K. Lohman[1,2], Aaron R. Quinlan[1,2,3+], Ryan M. Layer[1,2+]

1. Department of Human Genetics, University of Utah, Salt Lake City, UT

2. USTAR Center for Genetic Discovery, University of Utah, Salt Lake City, UT

3. Department of Biomedical Informatics, University of Utah, Salt Lake City, UT

+ *To whom correspondence should be addressed*

ABSTRACT

SV-plaudit is a framework for rapidly curating structural variant (SVs) predictions. For each SV, we generate an image that visualizes the coverage and alignment signals from a set of samples. Images are uploaded to our cloud framework where users assess the quality of each image using a client-side web application. Reports can then be generated as a tab-delimited file or annotated VCF. As a proof of principle, nine researchers collaborated for one hour to evaluate 1,350 SVs each. We anticipate that *SV-plaudit* will become a standard step in variant calling pipelines and the crowd-sourced curation of other biological results.

Code available at https://github.com/jbelyeu/SV-plaudit

Demonstration video available at https://www.youtube.com/watch?v=ono8kHMKxDs

KEYWORDS

Structural variants; Visualization; Manual curation

BACKGROUND

Large genomic rearrangements, or structural variants (SVs), are an abundant form of genetic variation within the human genome[1,2], and they play an important role in both species evolution[3,4] and human disease phenotypes[5–9]. While many methods have been developed to identify SVs from whole-genome sequencing (WGS) data[10–14], the accuracy of SV prediction remains far below that of single-nucleotide and insertion-deletion variants[1]. Improvements to SV detection algorithms have, in part, been limited by the availability and applicability of high-quality truth sets. While the Genome in a Bottle[15] consortium has made considerable progress toward a gold-standard variant truth set, the incredibly high quality of the data underlying this project (300X and PCR-free) calls into question the generality of the accuracy obtained in typical quality WGS datasets (30X with PCR-amplification).

Given the high false positive rate of SV calls from genome and exome sequencing, manual inspection is a critical quality control step, especially in clinical cases. Scrutiny of the evidence supporting an SV is considered to be a reliable "dry bench" validation technique, as the human eye can rapidly distinguish true SV signal from alignment artifacts. In principle, we could improve the accuracy of SV call sets by visually validating every variant. In practice, however, current genomic data visualization methods[16–21] were designed primarily for spot checking a small number of variants and are difficult to scale to the thousands of SVs in typical call sets. Therefore, a curated set of SVs requires a new framework that scales to thousands of SVs, minimizes the time needed to adjudicate individual variants, and manages the collective judgment of large and often geographically dispersed teams.

Here we present *SV-plaudit*, a fast, highly-scalable framework enabling teams of any size to collaborate on the rapid, web-based curation of thousands of SVs. In the web interface, users consider a curation question for a series of pre-computed images (**Fig 1**, **Supplementary Fig 1**) that contain the coverage, paired-end alignments, and split-read alignments for the region surrounding a candidate SV for a set of relevant samples (e.g., tumor and matched normal samples). The curation question is defined by the researcher to match the larger experimental design (e.g., a cancer study may ask if the variant a somatic variant, a germline variant, or

a false positive). Responses are collected and returned as a report which can be used to identify high-quality variants.

While a team of curators is not required, collecting multiple opinions for each SV allows *SV-plaudit* to report the consensus view (i.e., a "curation score") of each variant. This consensus is less susceptible to human error and does not require expert users to score variants. With *SV-plaudit*, it is practical to inspect and score every variant in a call set, thereby improving the accuracy of SV predictions in individual genomes, and curating high quality-truth sets for SV method tuning.

RESULTS

To assess *SV-plaudit's* utility for curating SVs, nine researchers in the Quinlan laboratory at the University of Utah manually inspected and scored the 1,350 SVs (1,310 deletions, 8 duplications, 4 insertions, and 28 inversions) that the 1000 Genomes Project[1] identified in the NA12878 genome (**Supplemental File 1**). Since we expect trio analysis to be a common use case of *SV-plaudit*, we included alignments from NA12878 and her parents (NA12891 and NA12892), and participants considered the curation question “The SV in the top sample (NA12878) is:” and answers “GOOD”, “BAD”, or “DE NOVO”. In total, the full experiment took less than two hours with Amazon costs totaling less than $0.05. The images (**Supplemental File 2**) were generated in 3 minutes (20 threads, 2.7 seconds per image) and uploading to S3 required 5 minutes (full command list in **Supplemental File 3**). The mean time to score all images was 60.1 minutes (2.67 seconds per image) (**Fig 2A,** reports in **Supplemental Files 4,5**). In the scoring process, no de novo variants were identified. 40 images did not render correctly due to issues in the alignment files (e.g., coverage gaps) and were removed from the subsequent analysis (**Supplemental File 6**).

For this experiment, we use a curation score that mapped “GOOD” and “DE NOVO” to the value one, “BAD” to the value zero, and the mean as the aggregation function (**Fig 2B**). Most (70.5%) of variants were scored unanimously, with 67.1% being unanimously “GOOD” (score = 1.0, e.g., **Fig 1A**) and 3.4% being unanimously “BAD” (score = 0.0, e.g. **Fig 1B**). Since we had nine scores for each variant, we expanded our definition of

"unambiguous" variants to be those with at most one dissenting vote (score <0.2 or >0.8), which accounts for 87.1% of the variants. The 12.9% of SVs that were "ambiguous" (more than one dissenting vote, 0.2<= score <=0.8) were generally small (median size of 310.5bp versus 899.5bp for all variants, **Fig 2C**) or contained conflicting evidence (e.g., paired-end and split-read evidence indicated an inversion and the read-depth evidence indicated a deletion, e.g., **Fig 1C.**).

Other methods, such as SVTYPER[22] and CNVNATOR[23], can independently assess the validity of SV calls. SVTYPER genotypes SVs for a given sample by comparing the number of discordant paired-end alignments and split-read alignments that support the SV to the number of pairs and reads that support the reference allele. CNVNATOR uses sequence coverage to estimate copy number for the region affected by the SV. Both of these methods confirm the voting results (**Fig 2D**). Considering the set of "unambiguous" deletions, SVTYPER and CNVNATOR agree with the *SV-plaudit* curation score in 92.3% and 81.7% of cases, respectively. Here, agreement means that unambiguous false SVs (curation score < 0.2) have a CNVNATOR copy number near two (between 1.4 and 2.4) or an SYTYPER genotype of homozygous reference. Unambiguous true SVs (curation score > 0.8) have a CNVNATOR copy number near one or zero (less than 1.4), or an SYTYPER genotype of non-reference (heterozygous or homozygous alternate).

Despite this consistency, using either SVTYPER or CNVNATOR to validate SVs can lead to false positives or false negatives. For example, CNVNATOR reported a copy number loss for 44.2% of the deletions that were scored as unanimously BAD, and SVTYPER called 30.7% of the deletions that were unanimously GOOD as homozygous reference. Conversely, CNVNATOR had few false negatives (2.4% of unanimously GOOD deletions were called as copy neutral), and SVTYPER had few false positives (0.2% of non-reference variants were unanimously BAD). This comparison is meant to demonstrate that different methods have distinct strengths and weaknesses, and should not be taken as a direct comparison between SVTYPER and CNVNATOR, since CNVNATOR was one of nine methods used by the 1000 Genomes project while SVYTPER was not.

These results demonstrate that, with *SV-plaudit*, manual curation can be a cost-effective and robust part of the SV detection process. While we anticipate that automated SV detection methods will continue to improve, due in part to the improved truth sets that *SV-plaudit* will provide, directly viewing SVs will remain an essential validation technique. By extending this validation to full call sets, *SV-plaudit* not only improves specificity but can also enhance sensitivity by allowing users to relax quality filters and rapidly screen large sets of calls. Beyond demonstrating *SV-plaudit's* utility, our curation of SVs for NA12878 is useful as a high-quality truth set for method development and tuning. A VCF of these variants annotated with their curation score is available in **Supplementary File 5**.

DISCUSSION

SV-plaudit is an efficient, scalable, and flexible framework for the manual curation of large-scale SV call sets. Backed by Amazon S3 and DynamoDB, *SV-plaudit* is easy to deploy and scales to teams of any size. Each instantiation of *SV-plaudit* is completely independent and can be deployed locally for private or sensitive datasets, or be distributed publicly to maximize participation. By rapidly providing a direct view of the raw data underlying candidate SVs, *SV-plaudit* delivers the infrastructure to manually inspect full SV call sets. *SV-plaudit* also allows researchers to specify the questions and answers that users consider to ensure that the curation outcome supports the larger experimental design. This functionality is vital to a wide range of WGS experiments, from method development to the interpretation of disease genomes. We are actively working on machine learning methods that will leverage the curation scores for thousands of SV predictions as training data.

CONCLUSIONS

SV-plaudit was designed to judge how well the data in an alignment file corroborate a candidate SV. The question of whether a particular SV is a false positive due to artifacts from sequencing or alignment is a broader issue that must be answered in the context of other data sources such as mappability and repeat annotations. While this second level of analysis is crucial, it is beyond the scope of this paper, and we argue this analysis be performed only for those SVs that are fully supported by the alignment data. While *SV-plaudit*

combines *samplot* and *PlotCritic* to enable the curation of structural variant images, we emphasize that the *PlotCritic* framework can be used to score images of any type. Therefore, we anticipate that this framework will facilitate "crowd-sourced" curation of many other biological images.

METHODS

Overview. *SV-plaudit* (**Fig 3**) is based on two software packages: ***samplot*** for SV image generation, and ***PlotCritic*** for staging the Amazon cloud environment and managing user input. Once the environment is staged, users log into the system and are presented with a series of SV images in either a random or predetermined order. For each image, the user answers the curation question and responses are logged. Reports on the progress of a project can be quickly generated at any point in the process.

***Samplot*.** *Samplot* is a Python program that uses *pysam*[24] to extract alignment data from a set of BAM or CRAM files, and *matplotlib*[25] to visualize the raw data for the genomic region surrounding a candidate SV (**Fig 3A)**. For each alignment file, *samplot* renders the depth of sequencing coverage, paired-end alignments, and split-read alignments where paired-end and split-read alignments are color-coded based by the type of SV they support (e.g., black for deletion, red for a duplication, etc.) (**Fig 1 Supplementary Figure 2,** which considers variants at different sequencing coverages, and **Supplementary Figure 3, which** depicts variants supported by long-read sequencing).[26,27] Alignments are positioned along the x-axis by genomic location and along the left y-axis by the distance between the ends (insert size), which helps users to differentiate normal alignments from discordant alignments that support an SV. Depth of sequencing coverage is also displayed on the right y-axis to allow users to inspect whether putative copy number changes are supported by the expected changes in coverage. To improve performance for large events, we downsample “normal” paired-end alignments (a +/- orientation and an insert size range that is within Z standard deviations from the mean; by default Z = 4). Plots for each alignment file are stacked and share a common x-axis that reports the chromosomal position. By convention, the sample of interest (e.g., proband or tumor) is displayed as the top track, followed by the set of related reference genomes tracks (e.g., parents and siblings, matched normal sample). Users may specify the exact order by using command line parameters to *samplot*. A visualization of genome annotations and genes

and exons within the locus is displayed below the alignment plots to provide context for assessing the SV's relevance to phenotypes. Rendering time depends on the number of samples, sequnce coverage, and the size of the SV, but most images will require less than 5 seconds, and *samplot* rendering can be parallelizable by SV call.

***PlotCritic*.** *PlotCritic* (**Fig 3B**) provides a simple web interface for scoring images and viewing reports that summarize the results from multiple users and SV images. *PlotCritic* is both highly scalable and easy to deploy. Images are stored on Amazon Web Services (AWS) S3 and DynamoDB tables store project configuration metadata and user responses. These AWS services allow *PlotCritic* to dynamically scale to any number of users. It also precludes the need for hosting a dedicated server, thereby facilitating deployment.

After *samplot* generates the SV images, *PlotCritic* manages their transfer to S3 and configures tables in DynamoDB based on a JSON configuration file (`config.json` file in **Fig 3B**). In this configuration file, one defines the curation questions posed to reviewers, as well as the allowed answers and associated keyboard bindings to allow faster responses (`curationQandA` field in **Fig 3B**). In turn, these dictate the text and buttons that appear on the resulting web interface. As such, it allows the interface to be easily customized to support a wide variety of curation scenarios. For example, a cancer experiment may display a tumor sample and matched normal sample and ask users if the SV appears in both samples (i.e., a germline variant) or just in the tumor sample (i.e., a somatic variant). To accomplish this, the curation question (`question` field in **Fig 3B**) could be “In which samples does the SV appear?”, and the answer options (`answers` field in **Fig 3B**) could be “TUMOR”, “BOTH”, “NORMAL”, “NEITHER”. Alternatively, in the case of a rare disease, the interface could display a proband and parents and ask if the SV is only in the proband (i.e., de novo) or if it is also in a parent (i.e., inherited). Since there is no limit to the length of a questions or number of answers options, *PlotCritic* can support more complex experimental scenarios.

Once results are collected, *PlotCritic* can generate a tab-delimited report or annotated VCF that, for each SV image, details the number of times the image was scored and the full set of answers it received. Additionally, a

curation score can be calculated for each image by providing a value for each answer option and an aggregation function (e.g., mean, median, mode, standard deviation, min, max). For example, consider the cancer example from above where the values three, two, one, and zero mapped to the answers “TUMOR”, “BOTH”, “NORMAL”, and “NEITHER”, respectively. If "mode" were selected as the curation function, then the curation score would reflect the opinion of a plurality of users. The mean would reflect the consensus among all users, and the standard deviation would capture the level of disagreement about each image. While we expect mean, median, mode, standard deviation, min, and max to satisfy most use cases, users can implement custom scores by operating on the tab-delimited reported.

Each *PlotCritic* project is protected by AWS Cognito user authentication, which securely restricts access to the project website to authenticated users. A project manager is the only authorized user at startup and can authenticate other users using Cognito’s secure services. The website can be further secured using HTTPS and additional controls, such as IP restrictions, can be put in place by configuring AWS IAM access controls directly for S3 and DynamoDB.

AVAILABILITY OF SOURCE CODE AND REQUIREMENTS

Project name: SV-Plaudit

Project home page: https://github.com/jbelyeu/SV-plaudit

Operating systems: Mac OS and Linux

Programing language: Python, bash

License: MIT

AVAILABILITY OF SUPPORTING DATA AND MATERIAL

The datasets generated and/or analyzed during the current study are available in the 1000 Genomes Project repository, ftp://ftp-trace.ncbi.nih.gov/1000genomes/ftp/phase3/data/

All data generated during this study are included in this published article and its supplementary information files.

DECLARATIONS

List of abbreviations

SV: Structural Variant

Ethics approval and consent to participate

Not applicable

Consent for publication

Not applicable

Competing interests

The authors declare that they have no competing interests.

Funding

This research was supported by a US National Human Genome Research Institute awards to RML (NIH K99HG009532) and ARQ (NIH R01HG006693 and NIH R01GM124355), as well as a US National Cancer Institute award to ARQ (NIH U24CA209999).

Authors' contributions

JRP and RML developed the software. JRB, TJN, BSP, TAS, JMH, SNK, MEC, BKL, and RML scored variants for the experiment. JRP, ARQ, and RML wrote the manuscript. ARQ and RML conceived the study.

REFERENCES

1. Sudmant, P. H. *et al.* An integrated map of structural variation in 2,504 human genomes. *Nature* **526,** 75–81 (2015).

2. Redon, R. *et al.* Global variation in copy number in the human genome. *Nature* **444,** 444–454 (2006).

3. Newman, T. L. *et al.* A genome-wide survey of structural variation between human and chimpanzee. *Genome Res.* **15,** 1344–1356 (2005).

4. Bailey, J. A. & Eichler, E. E. Primate segmental duplications: crucibles of evolution, diversity and disease. *Nat. Rev. Genet.* **7,** 552–564 (2006).

5. Payer, L. M. *et al.* Structural variants caused by Alu insertions are associated with risks for many human diseases. *Proc. Natl. Acad. Sci. U. S. A.* **114,** E3984–E3992 (2017).

6. Schubert, C. The genomic basis of the Williams-Beuren syndrome. *Cell. Mol. Life Sci.* **66,** 1178–1197 (2009).

7. Pleasance, E. D. *et al.* A comprehensive catalogue of somatic mutations from a human cancer genome. *Nature* **463,** 191–196 (2010).

8. Venkitaraman, A. R. Cancer susceptibility and the functions of BRCA1 and BRCA2. *Cell* **108,** 171–182 (2002).

9. Zhang, F., Gu, W., Hurles, M. E. & Lupski, J. R. Copy number variation in human health, disease, and evolution. *Annu. Rev. Genomics Hum. Genet.* **10,** 451–481 (2009).

10. Ye, K., Schulz, M. H., Long, Q., Apweiler, R. & Ning, Z. Pindel: a pattern growth approach to detect break points of large deletions and medium sized insertions from paired-end short reads. *Bioinformatics* **25,** 2865–2871 (2009).

11. Rausch, T. *et al.* DELLY: structural variant discovery by integrated paired-end and split-read analysis. *Bioinformatics* **28,** i333–i339 (2012).

12. Handsaker, R. E., Korn, J. M., Nemesh, J. & McCarroll, S. A. Discovery and genotyping of genome structural polymorphism by sequencing on a population scale. *Nat. Genet.* **43,** 269–276 (2011).

13. Kronenberg, Z. N. *et al.* Wham: Identifying Structural Variants of Biological Consequence. *PLoS Comput. Biol.* **11,** e1004572 (2015).

14. Layer, R. M., Chiang, C., Quinlan, A. R. & Hall, I. M. LUMPY: a probabilistic framework for structural

variant discovery. *Genome Biol.* **15,** R84 (2014).

15. Zook, J. M. *et al.* Integrating human sequence data sets provides a resource of benchmark SNP and indel genotype calls. *Nat. Biotechnol.* **32,** 246–251 (2014).
16. Thorvaldsdóttir, H., Robinson, J. T. & Mesirov, J. P. Integrative Genomics Viewer (IGV): high-performance genomics data visualization and exploration. *Brief. Bioinform.* **14,** 178–192 (2013).
17. Fiume, M., Williams, V., Brook, A. & Brudno, M. Savant: genome browser for high-throughput sequencing data. *Bioinformatics* **26,** 1938–1944 (2010).
18. Munro, J. E., Dunwoodie, S. L. & Giannoulatou, E. SVPV: a structural variant prediction viewer for paired-end sequencing datasets. *Bioinformatics* **33,** 2032–2033 (2017).
19. O'Brien, T. M., Ritz, A. M., Raphael, B. J. & Laidlaw, D. H. Gremlin: an interactive visualization model for analyzing genomic rearrangements. *IEEE Trans. Vis. Comput. Graph.* **16,** 918–926 (2010).
20. Wyczalkowski, M. A. *et al.* BreakPoint Surveyor: a pipeline for structural variant visualization. *Bioinformatics* **33,** 3121–3122 (2017).
21. Spies, N., Zook, J. M., Salit, M. & Sidow, A. svviz: a read viewer for validating structural variants. *Bioinformatics* **31,** 3994–3996 (2015).
22. Chiang, C. *et al.* SpeedSeq: ultra-fast personal genome analysis and interpretation. *Nat. Methods* **12,** 966–968 (2015).
23. Abyzov, A., Urban, A. E., Snyder, M. & Gerstein, M. CNVnator: an approach to discover, genotype, and characterize typical and atypical CNVs from family and population genome sequencing. *Genome Res.* **21,** 974–984 (2011).
24. [PDF]pysam documentation - Read the Docs.
25. Hunter, J. D. Matplotlib: A 2D Graphics Environment. *Computing in Science Engineering* **9,** 90–95 (2007).
26. Zook, J. M. *et al.* Data Descriptor: Extensive sequencing of seven human genomes to characterize benchmark reference materials. (2016). doi:10.1038/sdata.2016.25
27. Daniel Kortschak, R., S Pedersen, B. & L Adelson, D. bíogo/hts: high throughput sequence handling for the Go language. *JOSS* **2,** 168 (2017).

FIGURE LEGENDS

Figure 1. Example *samplot* images of putative deletion calls that were scored as **A**) unanimously GOOD, **B**) unanimously BAD, and **C**) ambiguous with a mix of GOOD and BAD scores with respect to the top sample (NA12878) in each plot. The black bar at the top of the figure indicates the genomic position of the predicted SV, and the following subfigures visualize the alignments and sequence coverage of each sample. Subplots report paired-end (square-ends connected by a solid line, annotated as concordant and discordant paired-end reads in **A**) and split-read (circle-ends connected by a dashed line, annotated in **A**) alignments by their genomic position (x-axis) and the distance between mapped ends (insert size, left y-axis). Colors indicate the type of event the alignment supports (black for deletion, red for duplication, and blue and green for inversion) and intensity indicates the concentration of alignments. The grey filled shapes report the sequence coverage distribution in the locus for each sample (right y-axis, annotated in **A**). The samples in each panel are a trio of father (NA12891), mother (NA12892), and daughter (NA12878).

Figure 2. A) The distribution of the time between when an image was presented and when it was scored. B) The distribution of curation scores. C) The SV size distribution for all, unanimous (score 0 or 1), unambiguous (score <0.2 or >0.8) and ambiguous (score >=0.2 and <= 0.8) variants. D) A comparison of predictions for deletions between CNVNATOR copy number calls (y-axis), SVTYPER genotypes (color, “Ref.” is homozygous reference and “Non-ref.” is heterozygous or homozygous alternate), and curation scores (x-axis). This demonstrates a general agreement between all methods with a concentration of reference genotypes and copy number two (no evidence for a deletion) at curation score less than 0.2, and non-reference and copy number one or zero events (evidence for a deletion) at curation score greater than 0.8. There are also false positives for CNVNATOR (copy number less than 2 at score = 0), and false negatives for SVTYPER (reference genotype at score = 1).

Figure 3. The *SV-Plaudit* process. **A**) *Samplot* generates an image for each SV from VCF considering a set of alignment (BAM or CRAM) files. **B**) *PlotCritic* uploads the images to an Amazon S3 bucket and prepares DynamoDB tables. Users select a curation answer (“GOOD”, “BAD”, or “DE NOVO”) for each SV image.

DynamoDB logs user responses and generates reports. Within a report, a curation score function can be specified by mapping answer options to values and selecting an aggregation function. Here “GOOD” and “DE NOVO” were mapped to one, “BAD” to zero, and the mean was used. One useful output option for a report is a VCF annotated with the curation scores (shown here in bold as a **SVP**).

Figure 1

Click here to download Figure Figure1.pdf

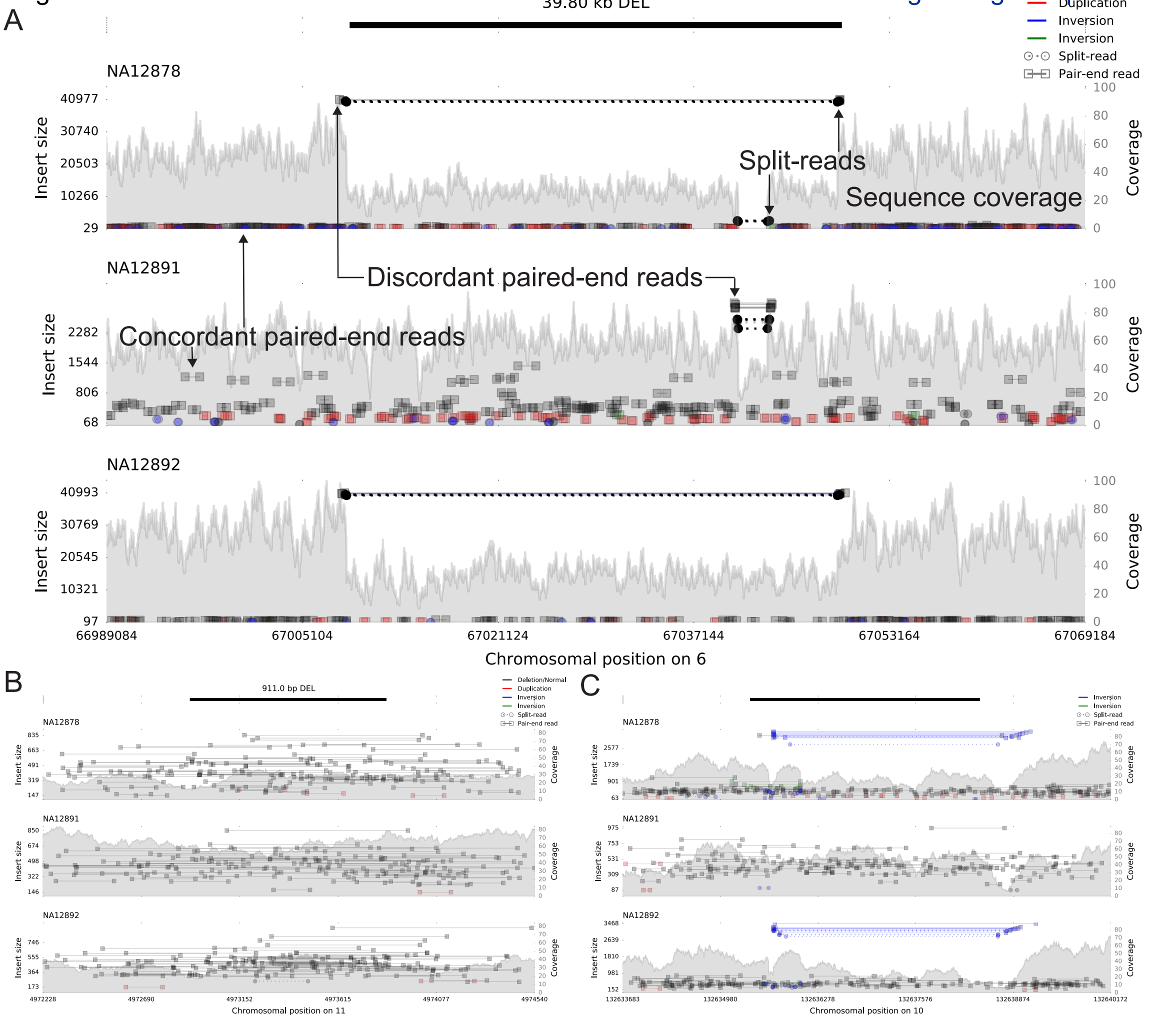

Figure 2

Click here to download Figure Figure2.pdf

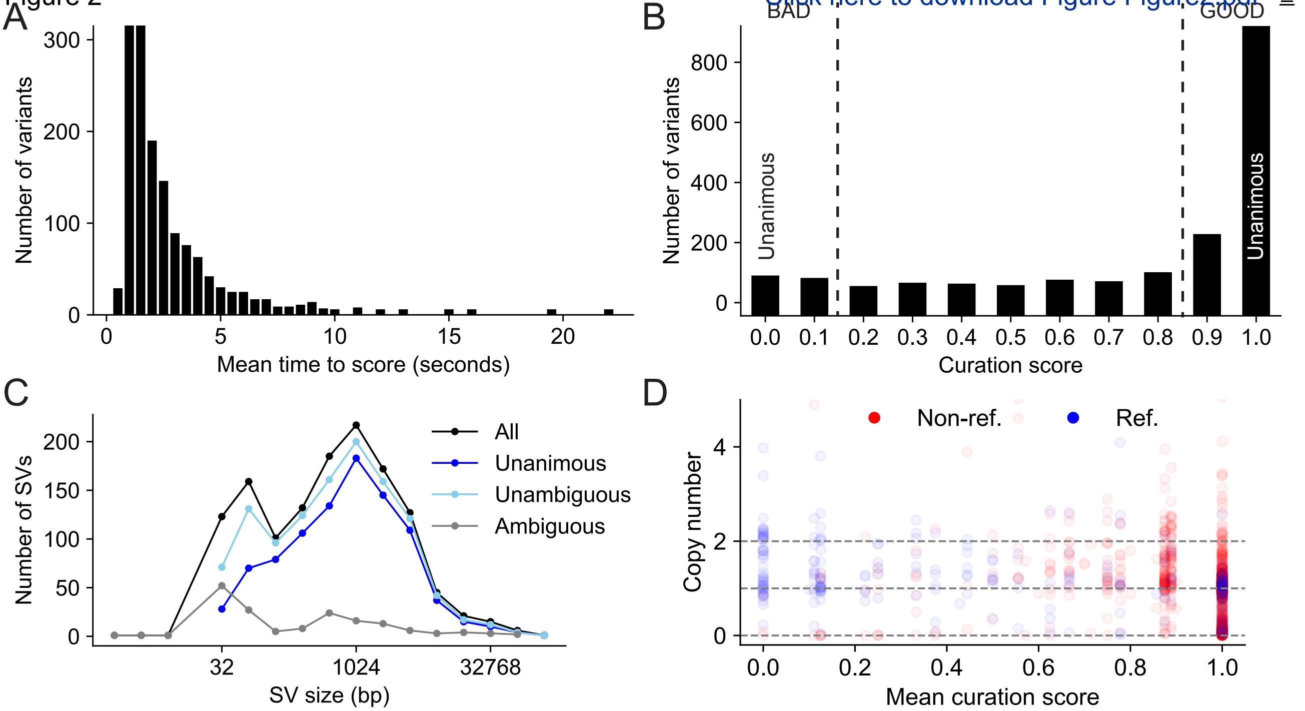

Figure 3

Click here to download Figure Figure3.pdf

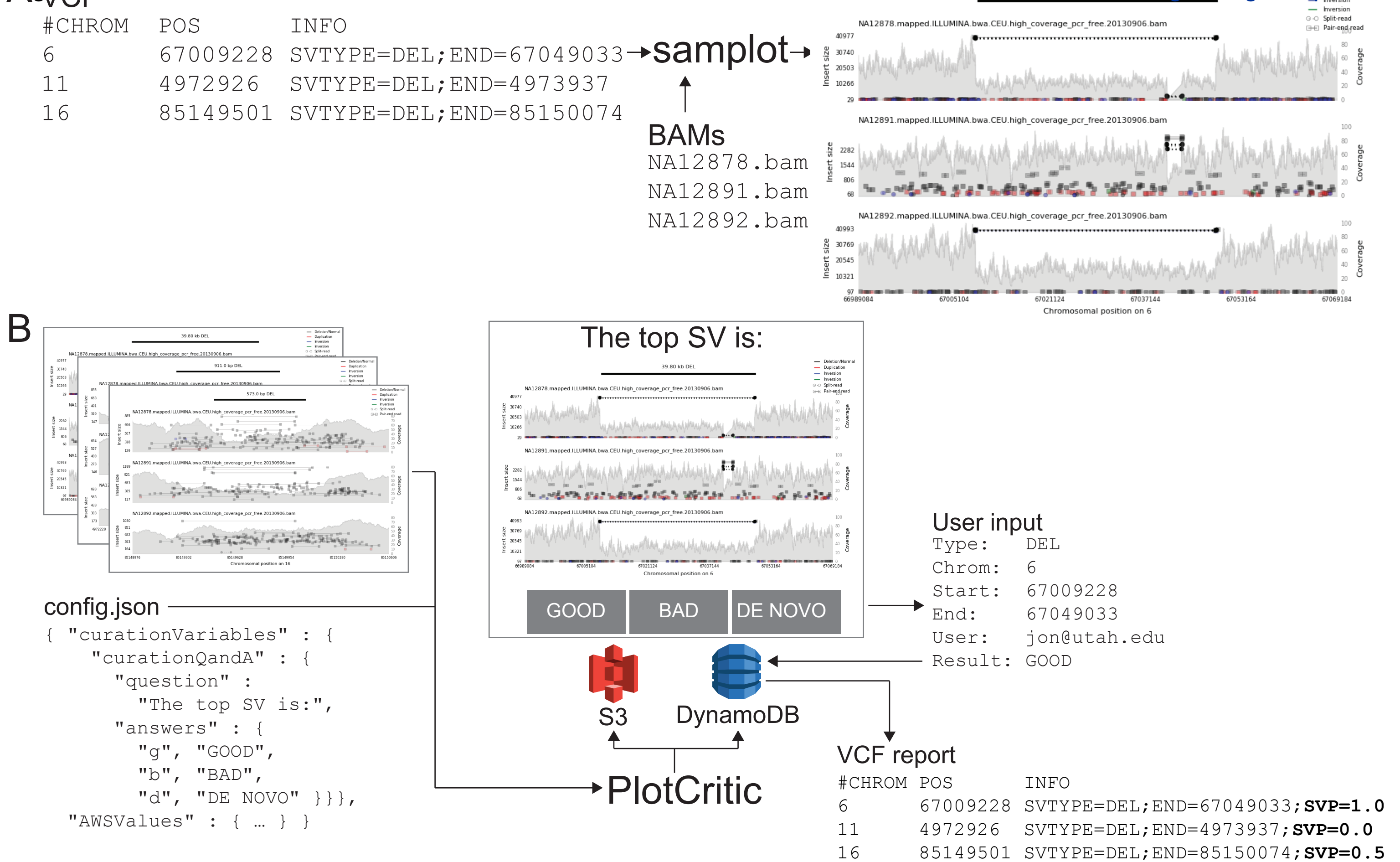

Supplementary File 1

Click here to access/download
Supplementary Material
Supplemental_File_1.vcf

Supplementary File 3

Click here to access/download
Supplementary Material
Supplemental_File_3.sh

Supplementary File 4

Click here to access/download
Supplementary Material
Supplemental_File_4.csv

Supplementary File 5

Click here to access/download
Supplementary Material
Supplemental_File_5.vcf

Click here to access/download
Supplementary Material
Supplemental_File_6.txt

Supplementary Figure 1

Click here to access/download
Supplementary Material
Supplemental_Figure_1.pdf

Supplementary Figure 2

Click here to access/download
Supplementary Material
Supplemental_Figure_2.pdf

Supplementary Figure 3

Click here to access/download
Supplementary Material
Supplemental_Figure_3.pdf
