# Supplementary material for: SV-plaudit: A cloud-based framework for manually curating thousands of structural variants
Source: Gigascience. 2018 May 31;7(7):giy064. doi: 10.1093/gigascience/giy064 (PMC6030999; doi:10.1093/gigascience/giy064)
Supplement: Additional Files [file giy064_supp.zip › Supplemental_Figure_1.pdf]

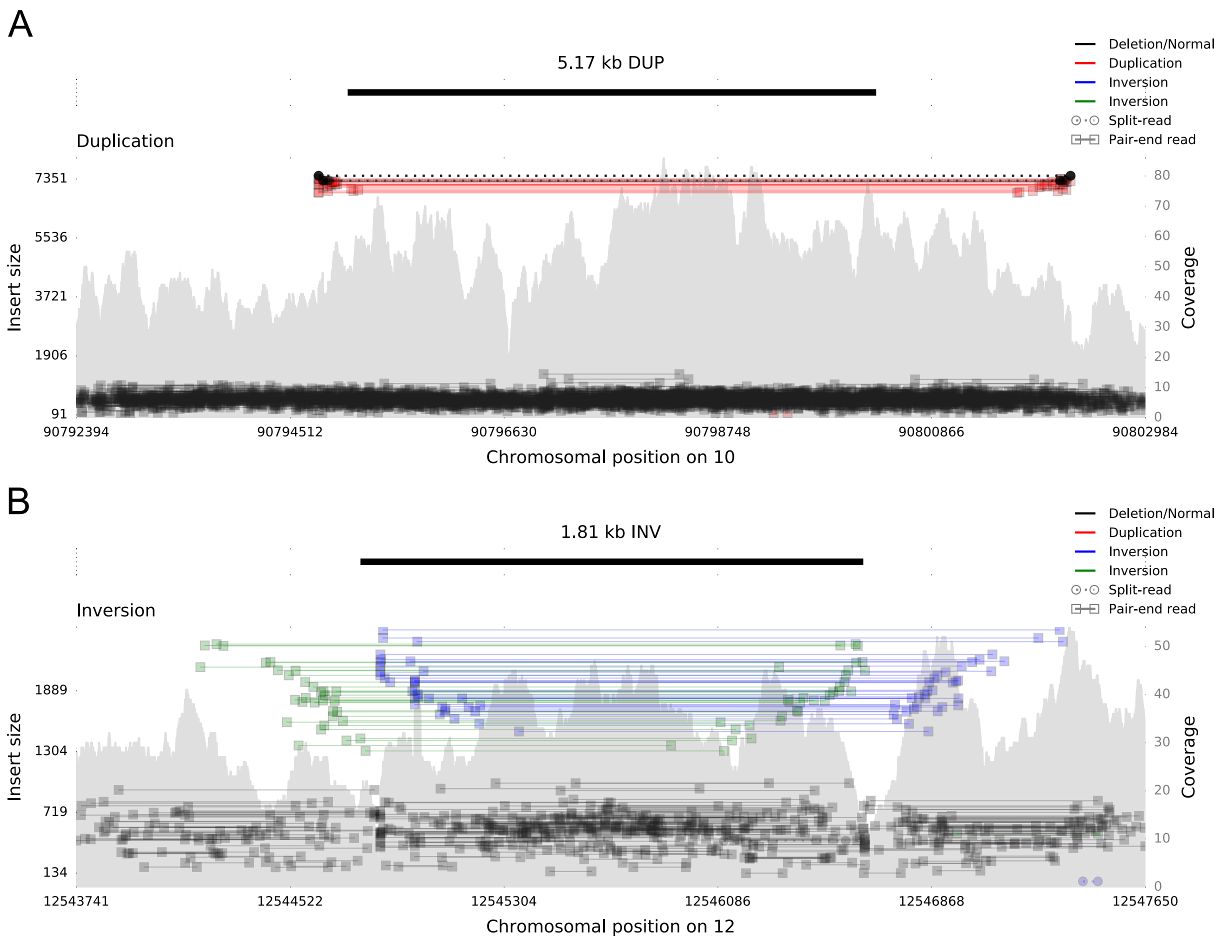

Supplemental Figure 1. Plots for different structural variant types shown in sample NA12878. **A**) A region is shown where a duplication event was called. **B**) A region is shown where an inversion event was called.
