# Supplementary material for: SV-plaudit: A cloud-based framework for manually curating thousands of structural variants
Source: Gigascience. 2018 May 31;7(7):giy064. doi: 10.1093/gigascience/giy064 (PMC6030999; doi:10.1093/gigascience/giy064)
Supplement: Additional Files [file giy064_supp.zip › Supplemental_Figure_2.pdf]

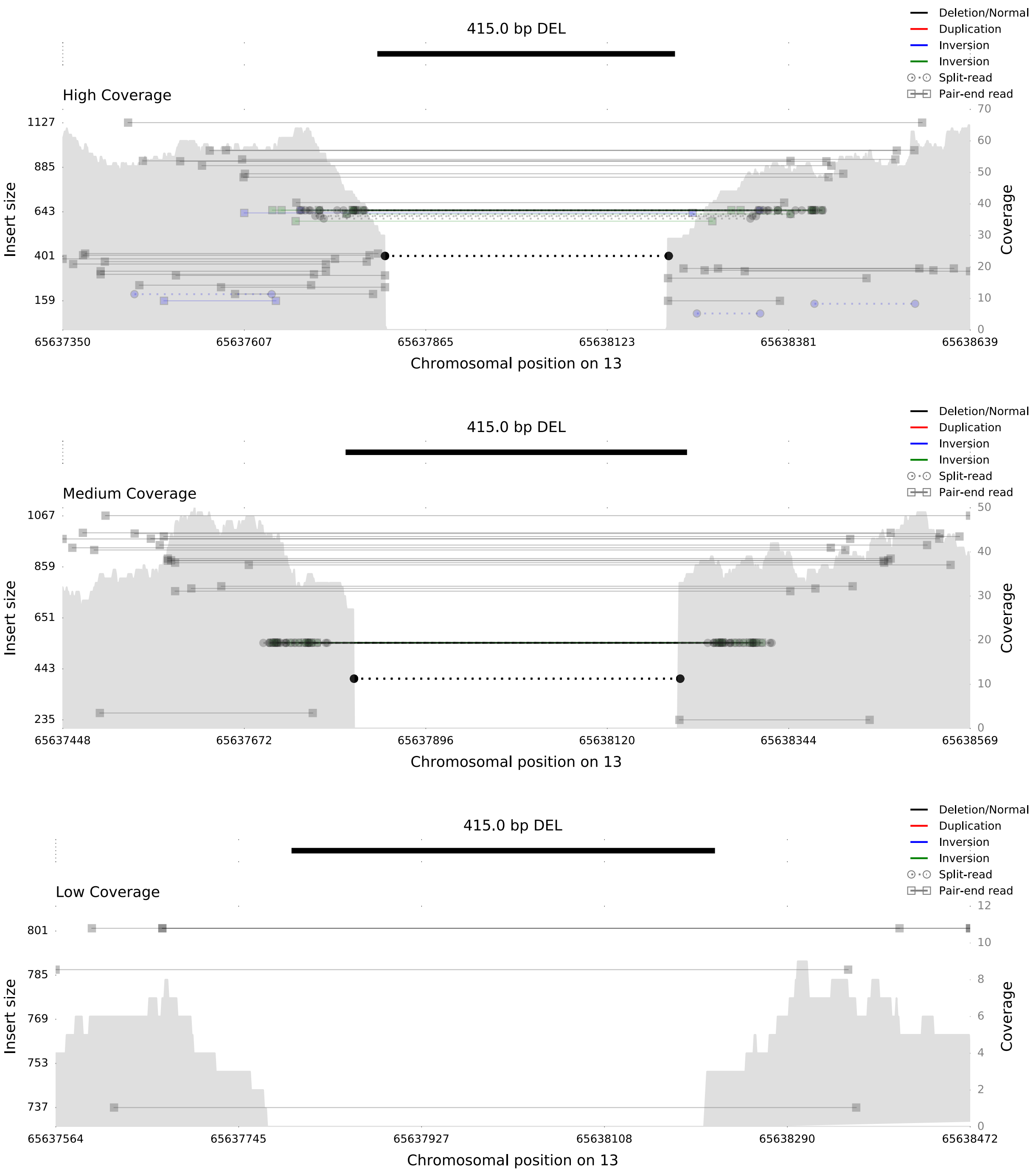

Supplemental Figure 2. A deletion call for sample NA12878 using different sequencing data to compare variant plots from high, medium, and low coverage levels. Mean sequencing depth of the BAM files used was **A**) 58X (1000 Genomes Project, high coverage), **B**) 33X (Genome in a Bottle Consortium), **C**) and 5X (1000 Genomes Project, low coverage).
