# Supplementary material for: SV-plaudit: A cloud-based framework for manually curating thousands of structural variants
Source: Gigascience. 2018 May 31;7(7):giy064. doi: 10.1093/gigascience/giy064 (PMC6030999; doi:10.1093/gigascience/giy064)
Supplement: Additional Files [file giy064_supp.zip › Supplemental_Figure_3.pdf]

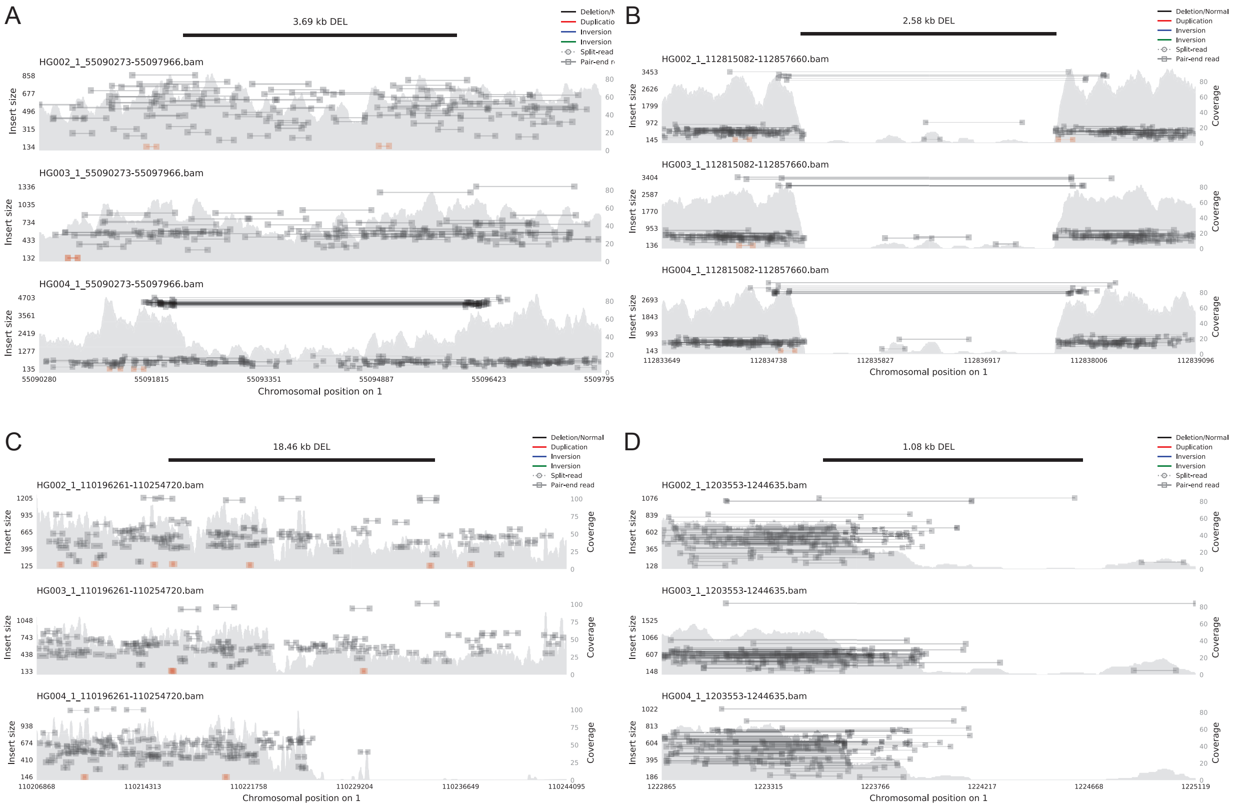

Supplemental Figure 3. A selection of structural variant visualizations from the Genome in a Bottle Ashkenazim trio version 0.5.0 call set and the NIST and NHGRI Illumina HiSeq samples with the FILGER field of the VCF set to “PASS” in **A**) and **B**), “LongReadHomRef” in **C**), and “NoConsensusGT” in **D**).
