# Supplementary material for: SV-plaudit: A cloud-based framework for manually curating thousands of structural variants
Source: Gigascience. 2018 May 31;7(7):giy064. doi: 10.1093/gigascience/giy064 (PMC6030999; doi:10.1093/gigascience/giy064)
Supplement: Response_to_Reviewer_Comments_Original_Submission.pdf [file giy064_response_to_reviewer_comments_original_submission.pdf]

Author's Response To Reviewer Comments

Close

Below are our point-by-point responses to all of issues raised by Reviewers 1 and 2. Our responses are wrapping in “----” markers.

Reviewer #1: The authors produce a tool that facilitates visual inspection of putative structural variants (i.e. deletions, inversions, duplications, insertions) based on reads mapped to a reference genome. The key innovation is that the software is set up so that a single researcher can rapidly visualize and categorize the existence of large numbers of putative structural variants. This enables a form of "crowd" evaluation such that every putative variant is visually inspected by multiple people. The software dramatically lowers the effort required to have manual inspection of manual curation of hundreds or even thousands of putative structural variants. This can lead to a strong increase in the reliability of putative SVs for downstream analyses and the development of new SV detection algorithms.

All the code is on Github with MIT license, the design of the software is modular for flexibility. This is pleasant.

I have not run the software, but the code and documentation appear to be functional, and the software uses standard input and output formats.

A weakness with the manuscript is that the software has only been tested on what the authors themselves call "the incredibly high quality" NA12878 genome in a bottle data (300x and PCR free), while also including the individual's parents. As the authors point out (L7-9), typical WGS datasets have been 30X coverage and with PCR-amplification during library preparation. There would thus be more power to evaluate the relevance of this software if PCR-biased, lower-coverage data were used (or simulated).

This is a good point. We have had success using SV-Plaudit on some internal sequencing experiments that were at 5X, 33X and 58X coverage. To help demonstrate this broader utility, we added Supplemental Figure 2 that shows an SV from NA12878 at these different coverage levels.

Some additional minor comments that could help to improve the manuscript & visualization:

1. the meaning of "GOOD" vs "BAD" vs "DE NOVO" is not immediately clear (e.g. L24 p3). And further appears to be at odds with the screenshots shown in the youtube video (Supports vs does not support vs de novo). Further more "de novo" is somewhat misleading as it suggest that something completely novel has occurred in the focal sample. Some efforts to make these buttons/meaning completely unambiguous would be justifiable. E.g., just have single statement: "Read mapping in the top image indicate that the sample has a xxx yyyy (e.g. 248bp DELETION) compared to the reference genome", then "TRUE", "FALSE" or "There appears to be a structural variant, but it differs from your suggestion". I also suspect that data could be cleaner if a fourth button existed to make it possible for users to say "I don't know".\

A strength of SV-Plaudit is that the “curation question” and “curation answers” are defined by the project manager, and one is free to easily customize the prompts to exactly fit your experiment. If there is a more efficient or less ambiguous way to prompt users, or if a third or fourth answer option is appropriate (e.g., a choice of “The region is too noisy” and “The region does not have adequate depth”), then one only needs to adjust the configuration file. We appreciate this feedback, as we do not think that we made this point clearly or strongly enough. While we discussed the details of how to customize the questions in the PlotCritic section of the methods, we also added more text addressing this issue in the discussion and background.
----

2. The manuscript takes putative SVs detected by the 1000 genomes project, evaluates them using SVTYPER users and then compares the results to those obtained using SVTYPER and CNVATOR. I suspect that SVTYPER and/or CNVATOR may have been used to create the initial putative SV dataset during the 1000G project. In which case this would be some circularity. A commentary on this would be welcome. Similarly, for those wanting to apply SVTYPER to a new genomic dataset, a recommendation on how to find putative SVs would be welcome.

This is not an issue that we considered and I thank the reviewer for bringing it to our attention. According to the 1000 genomes SV paper, CNVNATOR was used, and SVTYPER was not. Interestingly, the rate of false calls (false positives for CNVNATOR and false negatives for SVTYPER) was about the same for both methods (44.2% for CNVATOR and 30.7% for SVTYPER). When we go back and look at which algorithms were used in CNVNATOR’s false positives, they were all made by either a union of callers or by one of the other nine methods. We have added this commentary to the discussion of these results because it is another example of how every SV caller has its strengths and weaknesses, and why we believe visual validation is important.

As for adding text to the manuscript about using SVTYPER on a new dataset, we do not feel like we have the data to go beyond noting that in our experiment SVYTYPER marks some real deletions as homozygous reference. We hope that readers interpret the CNVNATOR and SVTYPER results as proof that it is difficult to rely on automated methods, and that visualization can help close the confidence gap for SVs.
----

3. Does PlotCritic have the option of hosting on a local machine, eg. using flask, instead of Amazon cloud? (for those with limited budgets, in places where AWS is difficult to access, and to cover for the situation where Amazon's API will change?)

This is a good point and we recognize this need. While Amazon hosting is all that is available right now, local hosting is actively being developed and will be available in a future release that we are planning for this year. Furthermore, Amazon provides an option to specify the API version desired for an application, which we use to maintain access and usability.
----

4. The screenshots and youtube video only appear to show DELETIONS. I would want to get a feeling for what duplications and insertions look like before using this software.

We agree that other SV types need to be shown and we have added Supplemental Figure 1, which includes a duplication and an inversion and updated the manuscript to refer to them.
----

5. Locations of read pair mappings may be clearer if there were no border on the pair of boxes and the line connecting the boxes were the same intensity as the boxes themselves (currently, the line goes from the middle of each square and is darker than the fill of the box)

We tried this and many other plot configurations, and we ultimately we decided that the current plots are most often the easiest to interpret. Thankfully the code is open source and advanced users can make small changes to the code to customize their plots. We have added comments in the code to make the appropriate lines easier to find and modify.
----

6. It took me a while to understand that the Y axis on each sample differed. Have you toyed with homogenising it? And/or perhaps showing it on a log scale?

Yes, we tried this and in our opinion it makes the plots less clear since the log transformation has the largest effect on the smallest insert size. We have also added the "--common_insert_size" option to samplot to use the same left y-scale across the plots.
----

7. Legend of Fig 1 might want to explicitly mention that NA12891 and 2 are parents of 12878. Furthermore it may want to mention that the top one is the one being evaluated.

Thank you. We have added this text to the figure.

Reviewer #2:

The authors of the manuscript "SV-plaudit: A cloud-based framework for manually curating thousands of structural variants" propose a framework to easily manually assess if SVs are potentially false or true. This is enabled based on a cloud based pipeline, which allows to look at multiple thousand sites for a larger community.
Overall I think that this is an important contribution for multiple projects such as GiaB or other where scientist need to assess the quality of their discovered SVs.

In the following some concerns and questions:

1. I am wondering if you could comment what had a deeper impact in the evaluation: a) the

visualization or b) the ability to look at the trio

Unfortunately, we do not know how the experiment participants felt because we did not ask. We expect that visualizing a trio would have a large impact on identifying true and false variants. We developed the tool around multi-sample visualization so that the users could get a sense of an SV's genomic context (e.g., is the area generally mess) from the control samples. A trio is helpful because users are able to observe both the genomic context and, in most cases, the inheritance of the SV.
----

2. I would encourage to include the mappability track of some kind (e.g. 36bp) to give the users more control and insight of the variability observed at the breakpoints. I know you stated that this needs to be part of a future research, but I think that is easy to obtain (UCSC) and integrate. Another maybe very useful feature would be the frequency of the reads that support the event.

This is a good point. Depending on the experiment, there are many types of annotations that users may want to see (e.g., repetitive elements, miRNAs, TAD boundaries). SAMPLOT has the option of displaying a gene annotation track. We have generalized this and exposed it to the SV-PLAUDIT configuration so that users can include any BED annotation (using the -A option) options.
----

3. I would encourage you to provide also figures for the other types of SVs not just Deletions. E.g. how do you visualize BND or other events?

We have added Supplementary Figure 1 that includes a duplication and an inversion, and BND visualization is under development.
----

4. I think your demonstration is really nice over the 1000 genomes data. What I would liked to see further is for some validated SVs if the figures are consistently clear. I know this is maybe out of the scope of this study, but maybe showing a few examples of the pass vs. non pass SVs from GiaB call set 0.5.0, which hopefully are close to the truth might give further insights on the reliability of the method. This is especially interesting since you mention false discovery and sensitivity issues over computational genotyping SVs.

We have added Supplementary Figure 3 based on the GiaB 0.5.0 call set and the GiaB/NIST/NHGRI Illumina sequencing of the Ashkenazim trio. The figure contains four panels, two SVs (A and B) were labeled PASS and two (C and D) were not labeled as PASS (LongReadHomRef and NoConsensusGT). The visualizations match the validation status of the VCF file.

The latest GiaB SV results
5. I found Figure 1 A rather confusing since I only see the coverage. Is this due to the size of

the region and thus the points on the bottom are the read pairs? In that case there should be some pairs that span the deletion, right? Could you maybe sort the reads better that support the SV, or more general show abnormal distances?

Figure 1A has a large cluster of both paired-end alignments (boxes and solid lines) and split-read alignments (circles and dashed lines) that appear to support the the SV in both NA12878 (top sample) and her dad. We have added some annotations to Figure 1A to make it easier to identify the different components.

Close
