# Supplementary material for: SV-plaudit: A cloud-based framework for manually curating thousands of structural variants
Source: Gigascience. 2018 May 31;7(7):giy064. doi: 10.1093/gigascience/giy064 (PMC6030999; doi:10.1093/gigascience/giy064)
Supplement: Response_to_Reviewer_Comments_Revision_1.pdf [file giy064_response_to_reviewer_comments_revision_1.pdf]

Author's Response To Reviewer Comments

Close

We added the citation for the data snapshot and RRID.

Close
