# Supplementary material for: SV-plaudit: A cloud-based framework for manually curating thousands of structural variants
Source: Gigascience. 2018 May 31;7(7):giy064. doi: 10.1093/gigascience/giy064 (PMC6030999; doi:10.1093/gigascience/giy064)
Supplement: Reviewer_1_Report_(Original_Submission) -- Yannick Wurm — 4/9/2018 Reviewed [file giy064_reviewer_1_report_(original_submission).pdf]

Reviewer Report

Title: **SV-plaudit: A cloud-based framework for manually curating thousands of structural variants**

Version: **Original Submission** **Date:** 4/9/2018

Reviewer name: **Yannick Wurm**

Reviewer Comments to Author: The authors produce a tool that facilitates visual inspection of putative structural variants (i.e. deletions, inversions, duplications, insertions) based on reads mapped to a reference genome. The key innovation is that the software is set up so that a single researcher can rapidly visualize and categorize the existence of large numbers of putative structural variants. This enables a form of "crowd" evaluation such that every putative variant is visually inspected by multiple people. The software dramatically lowers the effort required to have manual inspection of manual curation of hundreds or even thousands of putative structural variants. This can lead to a strong increase in the reliability of putative SVs for downstream analyses and the development of new SV detection algorithms.

All the code is on Github with MIT license, the design of the software is modular for flexibility. This is pleasant.

I have not run the software, but the code and documentation appear to be functional, and the software uses standard input and output formats.

A weakness with the manuscript is that the software has only been tested on what the authors themselves call "the incredibly high quality" NA12878 genome in a bottle data (300x and PCR free), while also including the individual's parents. As the authors point out (L7-9), typical WGS datasets have been 30X coverage and with PCR-amplification during library preparation. There would thus be more power to evaluate the relevance of this software if PCR-biased, lower-coverage data were used (or simulated).

Some additional minor comments that could help to improve the manuscript & visualization:

1. the meaning of "GOOD" vs "BAD" vs "DE NOVO" is not immediately clear (e.g. L24 p3). And further appears to be at odds with the screenshots shown in the youtube video (Supports vs does not support vs de novo). Further more "de novo" is somewhat misleading as it suggest that something completely novel has occurred in the focal sample. Some efforts to make these buttons/meaning completely unambiguous would be justifiable. E.g., just have single statement: "Read mapping in the top image indicate that the sample has a xxx yyyy (e.g. 248bp DELETION) compared to the reference genome", then "TRUE", "FALSE" or "There appears to be a structural variant, but it differs from your suggestion". I also suspect that data could be cleaner if a fourth button existed to make it possible for users to say "I don't know".

2. The manuscript takes putative SVs detected by the 1000 genomes project, evaluates them using SVTYPER users and then compares the results to those obtained using SVTYPER and CNVATOR. I suspect that SVTYPER and/or CNVATOR may have been used to create the initial putative SV dataset during the 1000G project. In which case this would be some circularity. A commentary on this would be welcome. Similarly, for those wanting to apply SVTYPER to a new genomic dataset, a recommendation on how to find putative SVs would be welcome.

3. Does PlotCritic have the option of hosting on a local machine, eg. using flask, instead of Amazon cloud? (for those with limited budgets, in places where AWS is difficult to access, and to cover for the situation

where Amazon's API will change?)

4. The screenshots and youtube video only appear to show DELETIONS. I would want to get a feeling for what duplications and insertions look like before using this software.

5. Locations of read pair mappings may be clearer if there were no border on the pair of boxes and the line connecting the boxes were the same intensity as the boxes themselves (currently, the line goes from the middle of each square and is darker than the fill of the box)

6. It took me a while to understand that the Y axis on each sample differed. Have you toyed with homogenising it? And/or perhaps showing it on a log scale?

7. Legend of Fig 1 might want to explicitly mention that NA12891 and 2 are parents of 12878. Furthermore it may want to mention that the top one is the one being evaluated.

Click here to enter text.

Methods

Are the methods appropriate to the aims of the study, are they well described, and are necessary controls included? Yes

Conclusions

Are the conclusions adequately supported by the data shown? Yes

Reporting Standards

Does the manuscript adhere to the journal's guidelines on minimum standards of reporting? No

Choose an item.

Statistics

Are you able to assess all statistics in the manuscript, including the appropriateness of statistical tests used? There are no statistics in the manuscript.

Quality of Written English

Please indicate the quality of language in the manuscript: Acceptable

Declaration of Competing Interests

Please complete a declaration of competing interests, considering the following questions:

- Have you in the past five years received reimbursements, fees, funding, or salary from an organisation that may in any way gain or lose financially from the publication of this manuscript, either now or in the future?

- Do you hold any stocks or shares in an organisation that may in any way gain or lose financially from the publication of this manuscript, either now or in the future?
- Do you hold or are you currently applying for any patents relating to the content of the manuscript?
- Have you received reimbursements, fees, funding, or salary from an organization that holds or has applied for patents relating to the content of the manuscript?
- Do you have any other financial competing interests?
- Do you have any non-financial competing interests in relation to this paper?

If you can answer no to all of the above, write 'I declare that I have no competing interests' below. If your reply is yes to any, please give details below.

I declare that I have no competing interests

I agree to the open peer review policy of the journal. I understand that my name will be included on my report to the authors and, if the manuscript is accepted for publication, my named report including any attachments I upload will be posted on the website along with the authors' responses. I agree for my report to be made available under an Open Access Creative Commons CC-BY license (http://creativecommons.org/licenses/by/4.0/). I understand that any comments which I do not wish to be included in my named report can be included as confidential comments to the editors, which will not be published.

I agree to the open peer review policy of the journal

To further support our reviewers, we have joined with Publons, where you can gain additional credit to further highlight your hard work (see: https://publons.com/journal/530/gigascience). On publication of this paper, your review will be automatically added to Publons, you can then choose whether or not to claim your Publons credit. I understand this statement.

Yes Choose an item.
