# Supplementary material for: SV-plaudit: A cloud-based framework for manually curating thousands of structural variants
Source: Gigascience. 2018 May 31;7(7):giy064. doi: 10.1093/gigascience/giy064 (PMC6030999; doi:10.1093/gigascience/giy064)
Supplement: Reviewer_2_Report_(Original_Submission) -- Fritz Sedlazeck — 4/9/2018 Reviewed [file giy064_reviewer_2_report_(original_submission).pdf]

**Reviewer Report**

**Title: SV-plaudit: A cloud-based framework for manually curating thousands of structural variants**

**Version: Original Submission** **Date:** 4/9/2018

**Reviewer name: Fritz Sedlazeck**

**Reviewer Comments to Author:**

The authors of the manuscript "SV-plaudit: A cloud-based framework for manually curating thousands of structural variants" propose a framework to easily manually assess if SVs are potentially false or true. This is enabled based on a cloud based pipeline, which allows to look at multiple thousand sites for a larger community.
Overall I think that this is an important contribution for multiple projects such as GiaB or other where scientist need to assess the quality of their discovered SVs.

In the following some concerns and questions:

1. I am wondering if you could comment what had a deeper impact in the evaluation: a) the visualization or b) the ability to look at the trio

2. I would encourage to include the mappability track of some kind (e.g. 36bp) to give the users more control and insight of the variability observed at the breakpoints. I know you stated that this needs to be part of a future research, but I think that is easy to obtain (UCSC) and integrate. Another maybe very useful feature would be the frequency of the reads that support the event.

3. I would encourage you to provide also figures for the other types of SVs not just Deletions. E.g. how do you visualize BND or other events?

4. I think your demonstration is really nice over the 1000 genomes data. What I would liked to see further is for some validated SVs if the figures are consistently clear. I know this is maybe out of the scope of this study, but maybe showing a few examples of the pass vs. non pass SVs from GiaB call set 0.5.0, which hopefully are close to the truth might give further insights on the reliability of the method. This is especially interesting since you mention false discovery and sensitivity issues over computational genotyping SVs.

5. I found Figure 1 A rather confusing since I only see the coverage. Is this due to the size of the region and thus the points on the bottom are the read pairs? In that case there should be some pairs that span the deletion, right? Could you maybe sort the reads better that support the SV, or more general show abnormal distances?

**Methods**

Are the methods appropriate to the aims of the study, are they well described, and are necessary controls included? Yes

**Conclusions**

Are the conclusions adequately supported by the data shown? Yes

**Reporting Standards**

Does the manuscript adhere to the journal's guidelines on minimum standards of reporting? Yes

Choose an item.

**Statistics**

Are you able to assess all statistics in the manuscript, including the appropriateness of statistical tests used? There are no statistics in the manuscript.

**Quality of Written English**

Please indicate the quality of language in the manuscript: Acceptable

**Declaration of Competing Interests**

Please complete a declaration of competing interests, considering the following questions:

- Have you in the past five years received reimbursements, fees, funding, or salary from an organisation that may in any way gain or lose financially from the publication of this manuscript, either now or in the future?
- Do you hold any stocks or shares in an organisation that may in any way gain or lose financially from the publication of this manuscript, either now or in the future?
- Do you hold or are you currently applying for any patents relating to the content of the manuscript?
- Have you received reimbursements, fees, funding, or salary from an organization that holds or has applied for patents relating to the content of the manuscript?
- Do you have any other financial competing interests?
- Do you have any non-financial competing interests in relation to this paper?

If you can answer no to all of the above, write 'I declare that I have no competing interests' below. If your reply is yes to any, please give details below.

I declare that I have no competing interests

I agree to the open peer review policy of the journal. I understand that my name will be included on my report to the authors and, if the manuscript is accepted for publication, my named report including any attachments I upload will be posted on the website along with the authors' responses. I agree for my report to be made available under an Open Access Creative Commons CC-BY license (http://creativecommons.org/licenses/by/4.0/). I understand that any comments which I do not wish to be included in my named report can be included as confidential comments to the editors, which will not be published.

I agree to the open peer review policy of the journal

To further support our reviewers, we have joined with Publons, where you can gain additional credit to further highlight your hard work (see: https://publons.com/journal/530/gigascience). On publication of this paper, your review will be automatically added to Publons, you can then choose whether or not to claim your Publons credit. I understand this statement.

Yes Choose an item.
